# Influence of freeze-thawed cycles on pork quality

**DOI:** 10.5713/ajas.20.0416

**Published:** 2020-11-03

**Authors:** Tiprawee Tippala, Nunyarat Koomkrong, Autchara Kayan

**Affiliations:** 1 Department of Animal Science, Faculty of Agriculture, Kasetsart University, Bangkok 10900, Thailand; 2 Department of Animal Science, Faculty of Science and Technology, Suratthani Rajabhat University, Suratthani 84100, Thailand

**Keywords:** Freeze-thawed Cycles, Pork Quality, Shear Force Value, Citrate Synthase, Muscle Fiber Characteristics

## Abstract

**Objective:**

This study was conducted to determine the effect of freeze-thawed cycles (Fresh meat, F-T 1 cycle and F-T 2 cycles) on the quality characteristics of porcine *longissimus dorsi* muscle.

**Methods:**

A total of 20 three-crossbred pigs (Duroc×[Large White×Landrace]) were randomly obtained from a commercial slaughterhouse in Thailand. Muscle samples were immediately taken from 10 to 11th of the *longissimus dorsi* for histochemical analysis. The muscles were cut into 2.54 cm-thick chops. A minimum of 20 chops were used for each treatment (fresh meat, freeze-thawed 1 and 2 cycles). Individually chops were packaged in polyethylene bags and frozen at −20°C for 6 months followed by thawing in refrigerator at 4°C for 24 h (the 1st freeze-thawed cycle). The freeze-thawed procedure was repeated for two cycles (the 2nd freeze-thawed cycle). Thawing loss, shear force value, citrate synthase activity and muscle fiber characteristics were determined on the muscles.

**Results:**

Results showed that increasing of freeze-thawed cycle increased the thawing loss (p<0.01) and citrate synthase activity (p<0.001). Shear force value of fresh meat was higher than freeze-thawed 1 and 2 cycles (F-T 1 cycle and F-T 2 cycles). Freeze-thawed cycles affected muscle characteristics. Muscle fiber area and muscle fiber diameter decreased with an increasing number of freeze-thawed cycles (p<0.001), while the thickness of endomysium and perimysium were increased (p<0.001).

**Conclusion:**

Repeated freeze-thawed cycles degraded muscle fiber structure and deteriorated pork quality.

## INTRODUCTION

Fresh meat is a perishable product due to its biological composition. Many factors influence the shelf life of meat such as storage temperature, atmospheric oxygen, endogenous enzymes, moisture, light and particularly, microorganisms [1]. Meat and meat products should be preserved by adequate technologies to maintain quality and safety. The principles of meat preservation are to inhibit microbial spoilage and extend the shelf life of meat [2]. One of the most commonly used methods for preserving meat and meat products is freezing [3]. In meat industry, the global meat export is currently worth more than US$ 13 billion and freezing plays an important role in ensuring meat safety being supplied to all regions of the world [4]. Nonetheless, freezing can deteriorate meat quality due to the formation, size and shape of ice crystals that affect the microstructure of frozen meat [5]. Generally, the excess amount of meat after thawing may be put in the freezer again, especially in restaurant and retail markets, these freeze-thawed cycles may be repeated several times [6]. The freeze-thawed cycles could also occur from the temperature fluctuation or abuse during storage, transportation, retail display and consumption [7]. Studies of effects of freeze-thawed cycles on the quality of meats such as chicken [8,9], beef [10], ovine [11], pork [12,13], shrimp [7,14], fish [15,16], sea cucumber [17], and rabbit [18] have reported that multiple freeze-thawed cycles could damage the overall textural and physicochemical quality of meat. Multiple freeze-thawed cycles induced repeated melting and reformation of ice crystals that caused damage of cell membrane and induced myofibrillar protein structural changes which resulted the loss of protein functionality and protein denaturation as well as protein aggregation. These will have affected the water-holding capacity and texture of meat [19]. The previous studied found that freeze-thawed cycles had influenced the water-holding capacity of porcine muscle. The results showed that the multiple freeze-thawed cycles increased the thawing loss of pork [12,20,21]. The muscle cell structure damaged by multiple freeze-thawed cycles caused the loss of muscle fiber integrity and muscle fiber shrinkage which related to the ability of muscle cells to retain water [13]. The cutting forces of pork increased after one cycle of freeze-thawed, but a further increase of freeze-thawed cycles would lead to a decrease of cutting force [12]. Furthermore, the multiple freeze-thawed cycles caused severe damage to muscle cell structure and organelles, leading to release of mitochondrial enzymes to the exudates [22,23]. Some specific enzymes and citrate synthase can be detected and used as markers to identify damage to frozen meat and chilled meat [24]. Citrate synthase is a mitochondrial enzyme that is released to the extracellular spaces when cells are damaged from ice crystallization during the freezing process [24]. The citrate synthase activity of fresh meat was lower than frozen meat [25]. Numerous studies have been studied the influence of repeated freeze-thawed cycles on physicochemical, biochemical and protein functionalities of *longissimus* muscles in pork [12,13,19]. However, using the histological and citrate synthase activity method for studying of the effect of freeze-thawed cycles are few. It has been recommended the maximal duration of storage for frozen pork be between 6 to 12 months [26]. There was an increasing water loss and peroxide value that occurred during 12 months of frozen storage [27]. The aim of this study was to investigate the effect of freeze-thawed cycles on the quality characteristics of porcine *longissimus dorsi* muscle by freezing pork for 6 months and repeated freeze-thawed for 2 cycles.

## MATERIALS AND METHODS

### Animal and muscle samples

A total of 20 three-crossbred pigs (Duroc×[Large White× Landrace]) were randomly obtained from a commercial slaughterhouse in Thailand. The pigs were slaughtered at approximately 6 months of age according to standard slaughtering procedures. The average slaughter weight was 106± 8.39 kilograms. After electrical stunning, carcasses were scalded, cleaned, eviscerated, and split. Muscle samples were immediately taken from 10 to 11th of the *longissimus dorsi* for histochemical analysis. The samples were kept in ice and transported to laboratory, and then stored at 4°C for 24 h. The fat and connective tissue were removed. The muscles were cut into 2.54 cm-thick chops. A minimum of 20 chops were used for each treatment (fresh meat, freeze-thawed 1 and 2 cycles). Individually chops were packaged in polyethylene bags and frozen at −20°C for 6 months followed by thawing in refrigerator at 4°C for 24 h (the 1st freeze-thawed cycle). The freeze-thawed procedure was repeated for two cycles (the 2nd freeze-thawed cycle).

### Drip loss and thawing loss analysis

Drip loss was analyzed based on a bag method. The *longissimus dorsi* muscle samples were cut in a size-standardized after 24 h post-mortem and then weighed. The samples were suspended in plastic bag, held at 4°C for 48 h, and thereafter reweighed. Drip loss was expressed as a percentage [28]. Thawing loss of the thawed samples were analyzed from the known weights of sample before and after thawing and expressed as a percentage [12].

### Shear force value

The samples (fresh meat, F-T 1 cycle and F-T 2 cycles meat) were kept in plastic bags and then boiled until the temperature of the sample core reached 75°C. From each cooked chop, 6 of 1.27 cm-diameter cores were cut parallel to the muscle fiber orientation. The shear force value of samples were measured using a texture analyzer (Stable Micro System, TA. XT plus, Surrey, England) with V-shape cutting blade. The crosshead speed of knife blade was 200 mm/min. The average of maximum force to cut transversally into of the sample was recorded as the shear force value (N) [29].

### Citrate synthase activity

The activity of citrate synthase was measured using Sigma Aldrich enzyme kit (Sigma-Aldrich, Schenelldorf, Germany) and a spectrophotometric method, that is based on the absorbance of a yellow product formed during the enzymatic reaction of 5-thio-2-nitrobenzoic acid (TNB) and CoA-S-S-TNB, when coenzyme A is released from acetyl-CoA and 5,5′-dithio-bis (2-nitrobenzoic) acid (DTNB) is added. The absorbance of TNB is measured at 412 nm in a glass cell (optical path length 10 mm). The samples from fresh meat, F-T 1 cycle and F-T 2 cycles meat were prepared as follows: 0.5 mL of the meat exudates and 10 μL of bicine buffer (N,N bis (2 hydroxyethyl) glycine) were mixed and diluted with demineralised water (1:9). Further, the sample was tempered to laboratory temperature (25°C) and 10 μL DTNB, 10 μL acetyl-CoA and 920 μL of the test solution for citrate synthase were added. First, the endogenic activity was determined after the sample was incubated for 20 seconds. After that, the measurement started and the absorbance was recorded every 10 seconds for the total time of 90 seconds. This was followed by the addition of 50 μL of oxaloacetate acid; the solution was incubated again for 20 seconds and the absorbance was measured again for 90 seconds, and the overall activity was estimated [24]. The final activity of citrate synthase was calculated according to the following formula:

$$\text{U }(\mu \text{mol}/\text{mL}/\text{min})=\frac{\Delta \text{A}412/\text{min}\times \text{V}\times \text{dil}}{\text{EmM}\times \text{L}\times \text{Venz}}$$

where: ΔA412/min = difference between the endogenic and overall activity of citrate synthase at 0 and 60 s; V = total volume = 1 mL (mL); dil = sample dilution; EmM = absorption coefficient TNB at 412 nm = 13.6 mM^−1^/cm; L = cell length = 1 cm; Venz = sample volume = 10 μL (mL).

### Histochemical analysis

The histological analysis was evaluated on the porcine *longissimus dorsi* muscle from fresh meat, F-T 1 cycle and F-T 2 cycles meat. The samples were cut into 0.5×0.5×1.0 cm pieces, then immediately fixed in 10% buffered neutral formalin solution for 24 h. After fixation, the specimens were dehydrated in alcohol, cleared in xylene, infiltrated, and finally embedded in paraffin. The sections were cut at 3 μm thickness and stained with hematoxylin and eosin for general histological study. Stained cross-sections were photographed with a light microscope (OlympusFSX100, Tokyo, Japan) at 10× objective lens and a 10× eye piece. Five photographs of different cross-sections from each muscle were taken. The samples were determined by using Image-J software (National Institute of Mental Health, Bethesda, MD, USA). The mean number of fibers per area was obtained by counting the total number of fibers (TNF) in five areas (each area: 582,007 μm^2^) per sample. The mean of approximately 300 fibers in five random fields for each muscle were measured to estimate the fiber diameters (μm) and fiber areas (μm^2^). The thickness of endomysium and perimysium were determined on each sample. The structural elements were measured in an area of fiber bundle. Forty measurements of the thickness (μm) of endomysium, and 10 measurements of the thickness (μm) of perimysium were made on each picture. The mean thickness was estimated from the measured values [30].

### Statistical analysis

Thawing loss data were analyzed by paired t-tests of SAS (SAS Inst. Inc., Cary, NC, USA). Values of p<0.05 were considered to indicate statistically significant differences between F-T 1 cycle and F-T 2 cycles. Shear force value, citrate synthase activity and muscle fiber characteristics were analyzed by analysis of variance (ANOVA) of SAS (SAS Inst. Inc., Cary, NC, USA). Values of p<0.05 were considered to indicate statistically significant differences between groups. Significant differences (p<0.05) among means were identified using Proc general linear model procedures. The results are presented as least squares means with standard errors.

## RESULTS AND DISCUSSION

### Drip loss and thawing loss

The results in this study showed that freeze-thawed cycles had affected on drip loss and thawing loss. Fresh meat had a drip loss of 3.88%. Thawing loss of the F-T 1 cycle group was 5.81%±0.50% and 7.72%±0.46% for F-T 2 cycles group (p< 0.01). Thawing loss of the F-T 1 cycle group was lower than the F-T 2 cycles group. Muscle contains approximately 75% of water. Much of water in the muscle is entrapped in the structure of cells, including intra- and extra-myofibrillar spaces. The mechanism of water-holding capacity is centered in proteins and structure, especially myofibrillar protein, that binds and entraps water. The previous study showed evidence that pH, ionic strength, and oxidation affected the ability of myofibrillar protein, myofibrils, and muscle cells to entrap water. The change in the intracellular structure of the muscle cells affected the ability of muscle cells to retain water [31]. Drip loss and thawing loss can be described as changes in the water-holding capacity of meat. The drip loss has a direct impact on meat weight and loss of proteins that influences economic loss to the meat industry, including decreased meat quality [32]. The identified drip loss groups could divided into a low group with drip loss <2.60%, medium group with drip loss 2.60% to 4.0% and high group with drip loss >4.0% [33]. The present study showed that fresh meat had a drip loss 3.88% and classified to the medium group of drip loss. Thawing loss of F-T 1 cycle was lower than the F-T 2 cycles group. The thawing loss increased as a number of freeze-thawed cycles increased [32]. Moreover, repeated freeze-thawed cycles had an influence on pork quality. The thawing loss of one cycle of freeze-thawed meat was 5.7% and increased to 9.0%, 11.7%, and 13.7% when the number of freeze-thawed cycles increased to 2, 3, and 4 cycles, respectively. The water loss of frozen meat might be due to the formation of ice crystal during freezing. The repeated freeze-thawed cycles induced repeated melting and reformation of ice crystals which caused disruption and damage to cell membranes, organelles and muscle structure [20]. Moreover, freeze-thawed cycles accelerated protein and lipid oxidation and changed myofibrillar protein as well as loss of myofibrillar protein functionality. The reduced protein functionality related to the loss of ability to entrap water of protein, lead to an increase of water loss [12]. Therefore, the multiple of freeze-thawed cycles decreased water-holding capacity, meat quality and consumer acceptability due to the loss of tasteful constituents such as some amino acids or nucleotides [8,14].

### Shear force values

**Figure 1:**
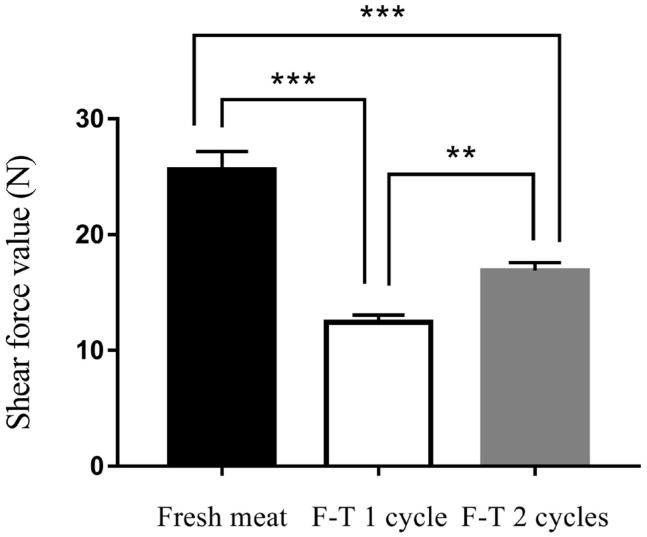
Shear force value of fresh meat, freeze-thawed 1 cycle (F-T 1 cycle) and freeze-thawed 2 cycles (F-T 2 cycles). Level of significance; ** p<0.01; *** p<0.001.

The results of present study showed that shear force value of fresh meat, F-T 1 cycle and F-T 2 cycles meats were 25.62± 1.04 N, 12.43±1.04 N, and 16.90±1.04 N, respectively (Figure 1). The shear force value of muscle was decreased after F-T 1 cycle. The F-T 2 cycles meat had an increased shear force value from 12.43±1.04 N to 16.90±1.04 N. The shear force value was measured as an objective measurement of meat tenderness. Tenderness is the eating quality trait that mostly affects consumer’s acceptance of meat [12]. The repeat freeze-thawed cycles had influenced shear force value. This study revealed that shear force value of muscle was decreased after F-T 1 cycle. The decreased of shear force value might be due to the loss of integrity of muscle fibers and connective tissue from repeat melting and ice crystal formation during freeze-thawed process [11,13,14]. The increased of number of freeze-thawed cycles accelerated protein and lipid oxidation which caused protein denaturation and lead to loss of protein functionality [8]. Furthermore, repeat freeze-thawed cycles induced the release of proteinase to extracellular spaces, leading to the hydrolysis of muscle proteins [34]. However, this study showed that the shear force value of F-T 2 cycles meat was increased from 12.43±1.04 N to 16.90±1.04 N. The increased of shear force value might be due to the loss of water after repeated freeze-thawed that affected on muscle fiber shrinkage [35]. Thus, the freeze-thawed cycles affected on eating quality of meat, resulted in a decrease in hardness, chewiness and resilience of meat [9,11].

### Citrate synthase activity

**Figure 2:**
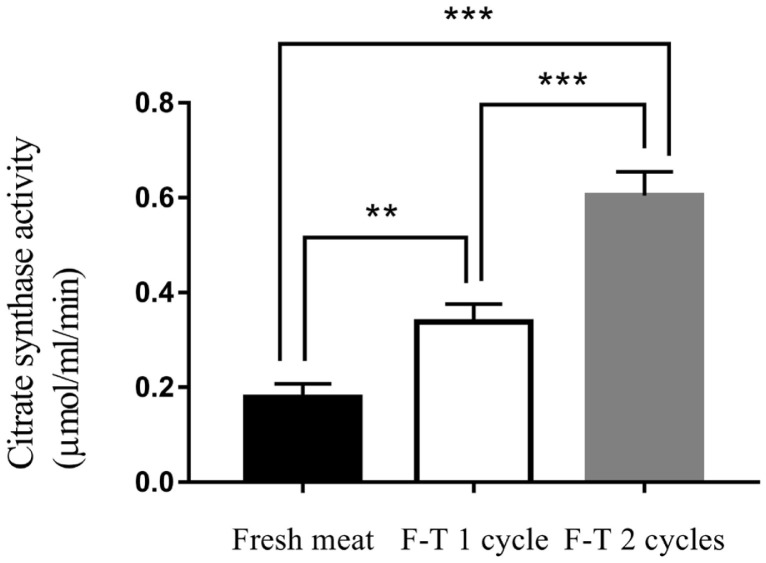
Citrate synthase activity of fresh meat, freeze-thawed 1 cycle (F-T 1 cycle) and freeze-thawed 2 cycles (F-T 2 cycles). Level of significance; ** p<0.01; *** p<0.001.

Freeze-thawed cycles affected citrate synthase activity. F-T 2 cycles meat had the highest level of citrate synthase activity (0.604±0.07 μmol/mL/min). Citrate synthase activity was decreased to 0.338±0.04 μmol/mL/min and 0.105±0.04 μmol/mL/min in F-T 1 cycle and fresh meat, respectively (Figure 2) (p<0.001). The citrate synthase activity of fresh meat was lower than frozen meat. The previous study reported that the activity of citrate synthase of chilled meat at 3 and 17 days were 0.196 and 0.319 μmol/mL/min, respectively. While the activity of citrate synthase of frozen meat at 3 and 17 days increased to 0.882 and 1.667 μmol/mL/min, respectively [25]. Citrate synthase activity increased in frozen chicken meat compared with fresh chicken meat. Citrate synthase is a mitochondrial enzyme that released to the extracellular spaces caused by cell damage from ice crystallization during freezing process and resulted in the increase of citrate synthase activity measurement [24]. Thus, freeze-thawed cycles were processes that affected on muscle cell and organelles integrity, leading to release of mitochondrial enzymes [22,23].

### Muscle fiber characteristics

**Figure 3:**
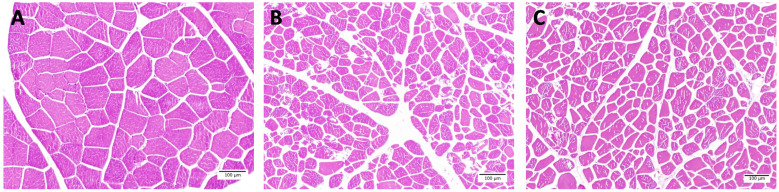
Muscle fiber characteristics of fresh meat (A), freeze-thawed 1 cycle (B) and freeze-thawed 2 cycles (C) (Scale bar = 100 μm).

This study showed that TNFs in fresh meat was less than F-T1 cycle and F-T 2 cycles meat (Table 1) (p<0.001). While muscle fiber area and diameter of fresh meat were larger than F-T 1 cycle and F-T 2 cycle meat, respectively (p<0.001). In addition, perimysium thickness of F-T 1 cycle and F-T 2 cycles meat were thicker than fresh meat (p<0.001). The endomysium thickness of F-T 2 cycles meat was higher than F-T 1 cycle and fresh meat, respectively (p<0.001). Light microscope images showed that repeated freeze-thawed cycles induced the rupture of muscle fibers and the appearance of endomysium thickness (Figure 3). The muscle fibers of fresh meat were intact, while the muscle fiber of F-T 1 cycle and F-T 2 cycles meat were torn and the spacing between muscle fibers increased as the number of freeze-thawed cycles increased. Muscle is approximately 75% water and water loss comes from water entrapped in the structure of muscle cells including the intracellular and extracellular spaces. Therefore, the changes of muscle cell structure is related to the ability of muscle cells to retain water [31]. The total fiber numbers has a negative relationship with the muscle fiber area, but has a positive relationship with drip loss, while the muscle fiber area has a negative relationship with drip loss [36]. The temperature of frozen meat at −22°C may cause the ice crystal formation located intra- and extracellular spaces. These ice crystals are large and irregular in shape. Ice crystals that are formed between fibers will generate pressure which separate fibers from each other, while ice crystals formed intracellular generated pressure in the opposite direction. Resulting from these opposite pressures, is an increased damage to the muscle fibers [37]. Freeze-thawed cycles were processes which caused the decrease of meat juiciness and texture [35]. The increasing of number of freeze-thawed cycles could cause the disintegration of muscle structure and decrease of muscle fiber diameter. The multiple freeze-thawed cycles induced the disruption of endomysium and caused a gap between muscle fibers [11]. Freeze-thawed cycles accelerated the protein and lipid oxidation, which lead to the muscle fiber shrinkage and the gap between muscle fiber being larger [12,38]. Furthermore, the repeated freeze-thawed cycles might affect other kinds of meat such as ovine [11], beef [10], chicken [9], prawn [39], fish [40], sea cucumber [17], and rabbit [18] .

In conclusion, the storage of pork meat in freezing condition for 6 months and then thawing influenced meat deterioration. Pork quality traits were significantly affected by repeated freeze-thawed cycles. Increasing of freeze-thawed cycles increased drip loss, thawing loss and citrate synthase activity and decreased shear force value. The repeated freeze-thawed cycles influenced muscle fiber characteristics. Freeze-thawed cycles were a process that influenced pork quality due to ice crystals causing damage to cell membranes, organelles, and muscle structure. Therefore, it is important to reduce freeze-thawed cycles and prevent temperature fluctuations during transportation and storage of meat to avoid the freeze–thawed cycles.

## Figures and Tables

**Table 1:** Effect of freeze-thawed cycles on muscle fiber characteristics (mean±standard error)

Traits	Fresh meat	F-T 1 cycle	F-T 2 cycles	p-value
TNF	123.01±5.62^b^	161.46±5.62^a^	151.07±5.62^a^	<0.001
CSA (μm^2^)	4,353.47±75.84^a^	2,189.82±75.84^b^	2,267.89±75.84^b^	<0.001
MFD (μm)	51.72±0.63^a^	36.65±0.63^b^	37.33±0.63^b^	<0.001
PT (μm)	11.58±0.44^b^	14.45±0.44^a^	15.30±0.44^a^	<0.001
ET (μm)	3.06±0.18^c^	4.12±0.18^b^	5.55±0.18^a^	<0.001

TNF, total number of fibers; CSA, cross-section area (μm^2^); MFD, muscle fiber diameter (μm); PT, perimysium thickness (μm); ET, endomysium thickness (μm).

a–c Means with different superscripts in the same row indicate a significant difference (p<0.05).
